# Real-World Outcomes of Ambulatory Drug-Susceptible Tuberculosis Management During the COVID-19 Pandemic in Romania

**DOI:** 10.3390/diseases14100370

**Published:** 2026-10-06

**Authors:** Ioana Munteanu, Alexandru Stoichiță, Silviu Gabriel Vlasceanu, Mara Balteanu, Oana Andreea Parlițeanu, Traian Constantin Panciu, Adriana Mihaela Iliesiu, Dragos Baiceanu, Ioana-Mădălina Moşteanu, Alexandru-Neculai Pavel, Beatrice Mahler

**Affiliations:** 1 Department of Pneumology, “Marius Nasta” National Institute of Pneumology, 050150 Bucharest, Romania; ioana.munteanu2015@yahoo.ro (I.M.); mara.balteanu@hotmail.com (M.B.); beatrice.mahler@umfcd.ro (B.M.); 2 Faculty of Medicine, Titu Maiorescu University, 040051 Bucharest, Romania; oana_andreea@yahoo.com; 3 Faculty of Medicine, University of Medicine and Pharmacy “Carol Davila”, 020021 Bucharest, Romania; silviu.vlasceanu@drd.umfcd.ro; 4 Department of Thoracic Surgery, “Marius Nasta” National Institute of Pneumology, 050150 Bucharest, Romania; 5 Department of Cardiology, “Th.Burghele” Clinical Hospital, 050653 Bucharest, Romania; 6 Doctoral School, University of Medicine and Pharmacy of Craiova, 200349 Craiova, Romania; 7 PAX Clinic, 020951 Bucharest, Romania

**Keywords:** tuberculosis, ambulatory care, COVID-19, pulmonary tuberculosis, patient-centered care, public health, respiratory medicine

## Abstract

Background: Romania continues to experience one of the highest tuberculosis (TB) burdens in the European Union/European Economic Area. During the COVID-19 pandemic, reduced inpatient capacity accelerated the use of ambulatory care for patients with drug-susceptible tuberculosis (DS-TB). This study evaluated treatment outcomes, outcome ascertainment, hospitalization requirements, and factors associated with unfavorable outcomes among patients managed through ambulatory TB services. Methods: We conducted a retrospective observational cohort study of adults with pulmonary or extrapulmonary DS-TB managed through ambulatory tuberculosis dispensaries affiliated with the “Marius Nasta” Institute of Pneumophthisiology, Bucharest, Romania, between January 2020 and December 2022. Treatment outcomes were defined according to World Health Organization criteria. Factors associated with unfavorable outcome were assessed using Firth penalized-likelihood logistic regression. Hospitalization during treatment was analyzed as a secondary outcome. Results: The final cohort comprised 545 patients with DS-TB, with a mean age of 44.8 years and 56.1% being male. Overall, 281 of 545 patients achieved treatment success, corresponding to a WHO-defined treatment-success rate of 51.6%. Twenty-seven patients (5.0%) experienced an unfavorable outcome, while 237 (43.5%) were classified as not evaluated. Among the 308 patients for whom a determinate end-of-treatment outcome was available, 281 (91.2%) achieved treatment success; however, this conditional proportion does not represent the programmatic treatment-success rate for the entire cohort. Conclusions: Structured ambulatory DS-TB care was operationally feasible within this tertiary-care-linked dispensary network during the COVID-19 pandemic. However, the WHO-defined treatment-success rate for the entire cohort was 51.6%, largely reflecting the substantial proportion of patients without an evaluable final outcome. The high proportion of not-evaluated patients represents an important programmatic weakness and limits conclusions regarding the effectiveness of the ambulatory model. Improved outcome ascertainment and continuity of follow-up are essential components of decentralized TB care.

## 1. Introduction

Tuberculosis (TB) remains a major public health challenge worldwide and continues to impose a substantial burden on healthcare systems, particularly in countries with persistent transmission and delayed case detection. Romania continues to report the highest TB notification rate in the European Union/European Economic Area, despite a progressive decline in TB incidence over the last decade [1]. Between 2008 and 2018, Romania recorded an average annual decrease in TB incidence of approximately 5.5%; however, the estimated case detection rate remained around 87%, indicating the need for further strengthening of diagnostic pathways, treatment access, and continuity of care [2,3].

Historically, TB care in Romania has relied predominantly on hospital-based management, especially at treatment initiation. This approach was traditionally supported by concerns related to transmission control, clinical monitoring, treatment adherence, and the management of patients with complex disease or social vulnerability. However, prolonged hospitalization may increase healthcare costs, disrupt patients’ social and professional lives, and contribute to TB-related stigma. In this context, international recommendations increasingly support patient-centered and decentralized models of care, including ambulatory management whenever clinically appropriate [4]. Such models aim to improve accessibility, preserve social and professional functioning, reduce unnecessary hospitalization, and maintain treatment effectiveness through structured outpatient monitoring and adherence support [5,6,7].

Before the COVID-19 pandemic, Romania had already initiated a gradual transition toward ambulatory TB care through health-system interventions supported by the ROU-T-MoH Transition Grant 1762, “Addressing health system-related challenges in TB care in Romania,” financed by the Global Fund to Fight AIDS, Tuberculosis and Malaria [3]. One of the main objectives of this initiative was to evaluate the feasibility of diagnosing, treating, and monitoring selected TB patients in outpatient TB dispensaries, while avoiding hospitalization when it was not clinically necessary.

The emergence of the COVID-19 pandemic in early 2020 profoundly disrupted healthcare delivery worldwide. In Romania, specialized pulmonology and TB hospitals were substantially involved in the management of patients with SARS-CoV-2 infection, reducing the availability of hospital beds traditionally dedicated to TB care. As a result, ambulatory TB management expanded rapidly, not only for smear-negative pulmonary TB cases, as initially intended in pilot interventions, but also for a broader spectrum of patients, including smear-positive pulmonary TB and extrapulmonary TB cases. Although this reorganization was primarily driven by pandemic-related pressure, it created an important opportunity to evaluate outpatient TB care under real-world conditions.

The “Marius Nasta” Institute of Pneumophthisiology in Bucharest is the largest tertiary referral center for respiratory diseases and TB management in Romania. During the pandemic, affiliated TB dispensaries played an expanded role in diagnosis, treatment initiation, follow-up, adherence monitoring, management of adverse drug reactions, and referral of patients requiring hospitalization. Evaluating the outcomes of patients managed through this model is therefore important for informing future TB policy and assessing whether ambulatory care can be safely and effectively integrated into routine TB services beyond the pandemic context.

Although Romania had already initiated a gradual transition toward ambulatory TB care before the COVID-19 pandemic, real-world evidence regarding the outcomes and safety of this model remains limited. The pandemic-related reduction in hospital capacity created an opportunity to evaluate ambulatory care for drug-susceptible tuberculosis under routine programmatic conditions. Therefore, the aim of the study was to assess treatment outcomes, hospitalization requirements, adverse drug reactions, diagnostic confirmation, treatment adherence, and predictors of unfavorable outcomes among patients with pulmonary or extrapulmonary drug-susceptible TB managed through ambulatory services affiliated with the “Marius Nasta” Institute of Pneumophthisiology between 2020 and 2022.

## 2. Materials and Methods

### 2.1. Study Design and Setting

We conducted a retrospective observational cohort study of adults with drug-susceptible tuberculosis (DS-TB) managed through the ambulatory tuberculosis dispensaries of Sectors 4, 5 and 6 of Bucharest, affiliated with the “Marius Nasta” Institute of Pneumophthisiology, Romania. The study covered patients who initiated ambulatory treatment between 1 January 2020 and 31 December 2022, during the COVID-19 pandemic.

### 2.2. Study Population and Cohort Definition

Of 561 patients included, 16 were excluded because the tuberculosis diagnosis was subsequently invalidated (non-TB), yielding a final DS-TB cohort of 545 patients. Restricting enrolment to 2020–2022 ensured that every included patient had at least 12 months for end-of-treatment outcome ascertainment, consistent with the WHO cohort-reporting convention of assigning outcomes one year after the last enrolment.

For the analysis of factors associated with treatment outcome, the cohort was restricted to patients with a determinate end-of-treatment outcome (treatment success or unfavourable outcome). Patients classified as “not evaluated” (see Section 2.3) were, by definition, without a final favourable/unfavourable status and were therefore excluded from the outcome regression while being fully described in the cohort characterization.

#### Inclusion and Exclusion Criteria

Patients were eligible for inclusion if they were ≥18 years old, had signed written informed consent, and were diagnosed with pulmonary or extrapulmonary DS-TB according to the criteria of the National Tuberculosis Prevention, Surveillance and Control Program. Eligible patients were registered in the tuberculosis dispensaries affiliated with the “Marius Nasta” Institute of Pneumophthisiology, and were managed through the ambulatory tuberculosis care program between January 2020 and December 2022.

Both bacteriologically confirmed and clinically diagnosed TB cases were included, provided that sufficient demographic, clinical, diagnostic, treatment-related, and outcome data were available in the TB registry or associated medical records. The descriptive cohort included all eligible adult patients registered in the ambulatory care program during the study period.

Patients were excluded from the descriptive cohort if they were <18 years old, had duplicate records, had an invalidated TB diagnosis, or had insufficient core clinical information preventing confirmation of TB diagnosis, registration status, or ambulatory management.

For binary treatment outcome analyses, patients with missing final treatment outcome data were excluded. Treatment success was defined as cure or treatment completion, whereas unfavorable treatment outcome was defined as treatment failure, death, treatment abandonment, or loss to follow-up. Therefore, treatment outcome analyses were restricted to patients with clearly classified final outcomes.

For diagnostic sub-analyses, including GeneXpert MTB/RIF testing, smear microscopy, and culture confirmation, patients were included only when the corresponding test result was available. Denominators differed according to data availability. The full descriptive cohort included 545 patients. Treatment-outcome analyses were restricted to the 308 patients with a determinate end-of-treatment outcome. Diagnostic analyses used variable-specific denominators according to test availability, and the corresponding denominator is reported for each analysis.

### 2.3. Ambulatory Care Model

Ambulatory TB care was delivered through TB dispensaries affiliated with the “Marius Nasta” Institute of Pneumophthisiology. Patients were managed as outpatients when hospitalization was not considered clinically mandatory according to National Tuberculosis Program procedures and treating physician’s assessment. The ambulatory model included treatment initiation or continuation, directly observed or supervised treatment according to national practice, scheduled clinical follow-up, monitoring of treatment adherence, identification and management of adverse drug reactions, and referral for hospitalization was made in cases of clinical deterioration, severe adverse drug reactions, complications, or other medical indications.

### 2.4. Data Sources and Variables

#### 2.4.1. Outcome Definitions

End-of-treatment outcomes were assigned according to the revised World Health Organization (WHO) treatment-outcome definitions published in 2021, which were proposed for use across all forms of TB and treatment regimens [1,2,3]. Cured and treatment completed together constitute treatment success. Unfavourable outcome comprised treatment failure, death during treatment, and loss to follow-up (treatment interrupted for ≥2 consecutive months); patients recorded as having taken part of the course and then stopped were classified as loss to follow-up. Patients with no assigned end-of-treatment outcome—including those transferred to another unit and those still on treatment were classified as not evaluated. Patients whose diagnosis was invalidated were not considered TB cases and were excluded entirely [1,2]. For patients transferred to another treatment unit, the final outcome was considered not evaluated when it could not be ascertained from the available registry or medical-record data; no outcome was imputed in these cases.

#### 2.4.2. Variables

Baseline variables recorded at treatment initiation were sex, age, area of residence (urban/rural), disease localization (pulmonary, extrapulmonary, or concurrent), sputum-smear microscopy status, GeneXpert MTB/RIF result, and rifampicin-resistance status. Treatment-process variables (documented non-compliance, adverse drug reactions, and hospitalization during treatment) were recorded but, because they occur during rather than before treatment, were analyzed descriptively rather than as baseline predictors.

#### 2.4.3. Statistical Analysis

Continuous variables are summarized as means with standard deviations or medians with interquartile ranges; categorical variables as counts and percentages, with denominators reported for each variable because of variable data completeness. Treatment success was defined according to the WHO programmatic indicator as the proportion of the entire eligible treatment cohort whose outcome was classified as cured or treatment completed. Accordingly, patients classified as not evaluated remained in the denominator but did not contribute to the treatment-success numerator. The primary treatment-success rate was therefore calculated as the number of patients cured or completing treatment divided by the full DS-TB cohort. The proportion of treatment success among patients with an assigned determinate outcome was additionally reported descriptively but was not considered the overall programmatic treatment-success rate. To assess whether missingness was informative, patients with a determinate outcome were compared with not-evaluated patients across baseline variables using the Mann–Whitney U test for age and Fisher’s exact test for categorical variables.

Factors associated with unfavourable outcome were examined using Firth’s penalized-likelihood logistic regression, which reduces small-sample bias and handles separation that ordinary logistic regression cannot; odds ratios (ORs) are reported with 95% profile-likelihood confidence intervals [8,9]. Given the limited number of unfavourable events, each baseline predictor was first screened univariately, and a parsimonious multivariable model was fitted with no more than two predictors to respect the events-per-variable constraint. GeneXpert MTB/RIF status was selected as the single adjustment covariate because it represented a clinically relevant baseline microbiological characteristic and had greater data completeness than smear microscopy, thereby limiting additional loss of observations in this event-limited analysis. The multivariable model included sex and GeneXpert MTB/RIF status. GeneXpert status was selected as the single adjustment covariate because it represented a clinically relevant baseline microbiological characteristic and had greater data completeness than smear microscopy, thereby limiting additional loss of observations in an already event-limited analysis. The model was intended as a parsimonious adjusted analysis rather than as a comprehensive prognostic model. A pre-specified sensitivity analysis excluded deaths from the unfavourable composite, because death is a competing event whose predictors may differ from those of treatment failure or loss to follow-up [10]. Hospitalization during treatment was analyzed as a secondary outcome. A two-sided *p* < 0.05 was considered significant. Analyses were performed using IBM SPSS Statistics, version 32.0.0 (IBM Corp., Armonk, NY, USA), and R, version 4.6.1 (R Foundation for Statistical Computing, Vienna, Austria). Firth penalized-likelihood logistic regression with profile-likelihood confidence intervals was implemented in R using the logistf package.

## 3. Results

### 3.1. Cohort Characteristics

**Figure 1:**
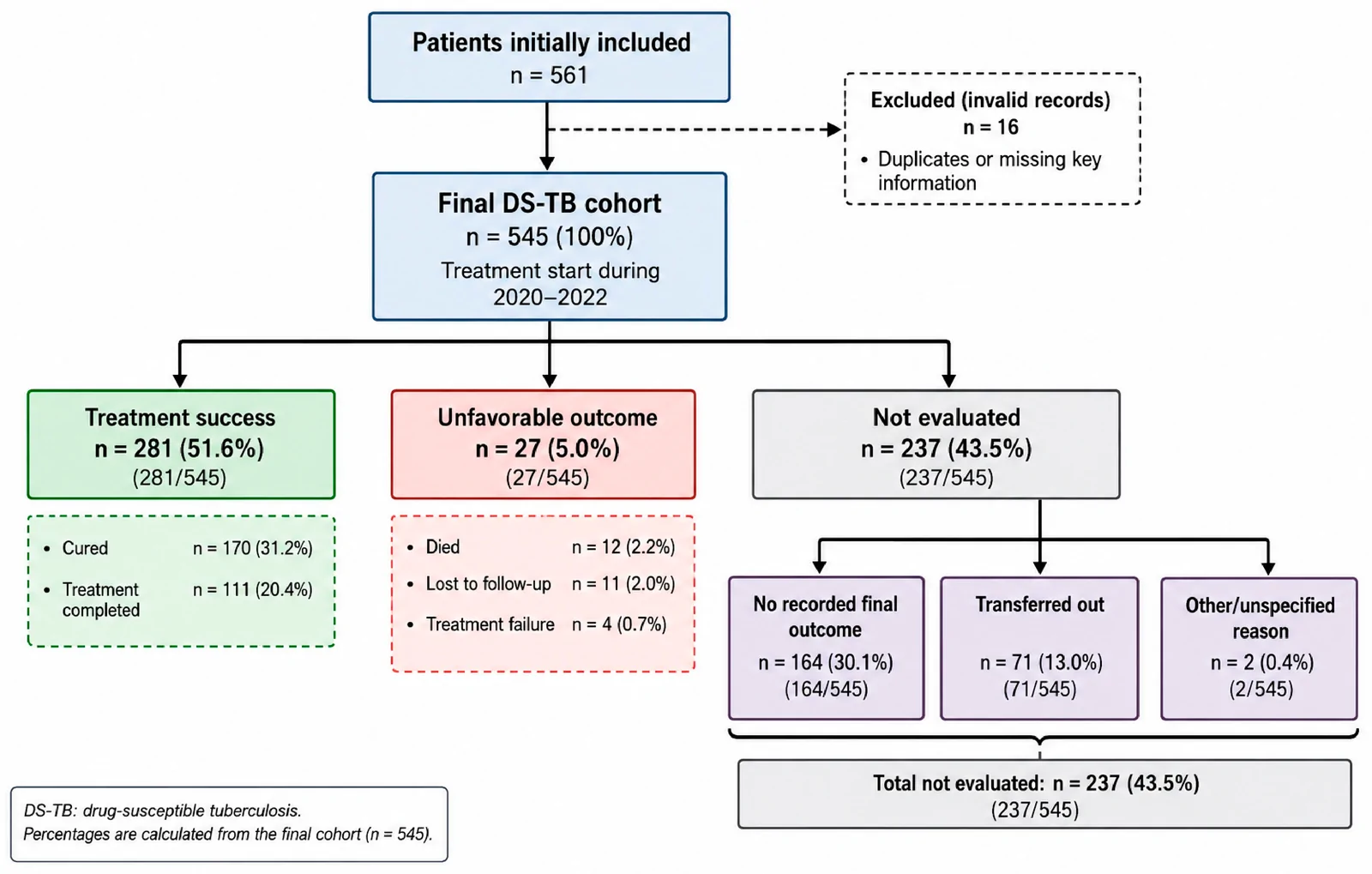
Cohort flow and end-of-treatment outcome classification (WHO 2021 definitions).

The final cohort comprised 545 patients with drug-susceptible tuberculosis. The mean age was 44.8 years (SD 16.0; median 42), and 306 (56.1%) were male. Most patients resided in urban areas, N = 443 (81.6% of those with residence recorded). Pulmonary disease predominated, N = 278 (80.6% of 345 with localization recorded), with extrapulmonary disease in N = 61 (17.7%) and concurrent disease in N = 6 (1.7%). Where microbiological results were available, 183 (52.1%) of 351 tested were smear-positive and 278 (68.6%) of 405 were GeneXpert-positive. Documented non-compliance, adverse drug reactions and hospitalization occurred in 67, 71 and 24 patients, respectively. Baseline characteristics are shown in Table 1, and the cohort flow in Figure 1.

### 3.2. Treatment Outcomes and Outcome Ascertainment

Among the 545 patients in the DS-TB cohort, 281 achieved treatment success (164 cured and 117 completed treatment), corresponding to an overall WHO-defined treatment-success rate of 51.6%. Twenty-seven patients (5.0%) had an unfavorable outcome, including 17 lost to follow-up, 5 deaths, and 5 treatment failures. A further 237 patients (43.5%) were classified as not evaluated. Among these, 164 had no recorded final treatment outcome, 71 had been transferred to another treatment unit without an ascertainable final outcome in the available records, and 2 were classified as not evaluated for other or unspecified reasons. Among the 308 patients with a determinate end-of-treatment outcome, 281 (91.2%) achieved treatment success. This latter proportion describes outcomes among patients with complete outcome ascertainment and should not be interpreted as the programmatic treatment-success rate for the entire cohort (Table 2).

### 3.3. Comparison of Evaluated and Not-Evaluated Patients

Patients with a determinate outcome differed from not-evaluated patients principally in disease localization: extrapulmonary disease was more frequent among not-evaluated patients (24% vs. 13%, *p* = 0.007), consistent with the greater likelihood of transfer or incomplete follow-up in extrapulmonary tuberculosis. Differences in age (median 41 vs. 44 years, *p* = 0.056), male sex (59% vs. 52%, *p* = 0.082) and GeneXpert positivity (65% vs. 74%, *p* = 0.081) were not statistically significant, while rural residence and smear positivity were similar (Table 3). This indicates the missingness was at least partly informative and should temper interpretation of the complete-case success rate.

### 3.4. Factors Associated with Unfavourable Outcome

**Figure 2:**
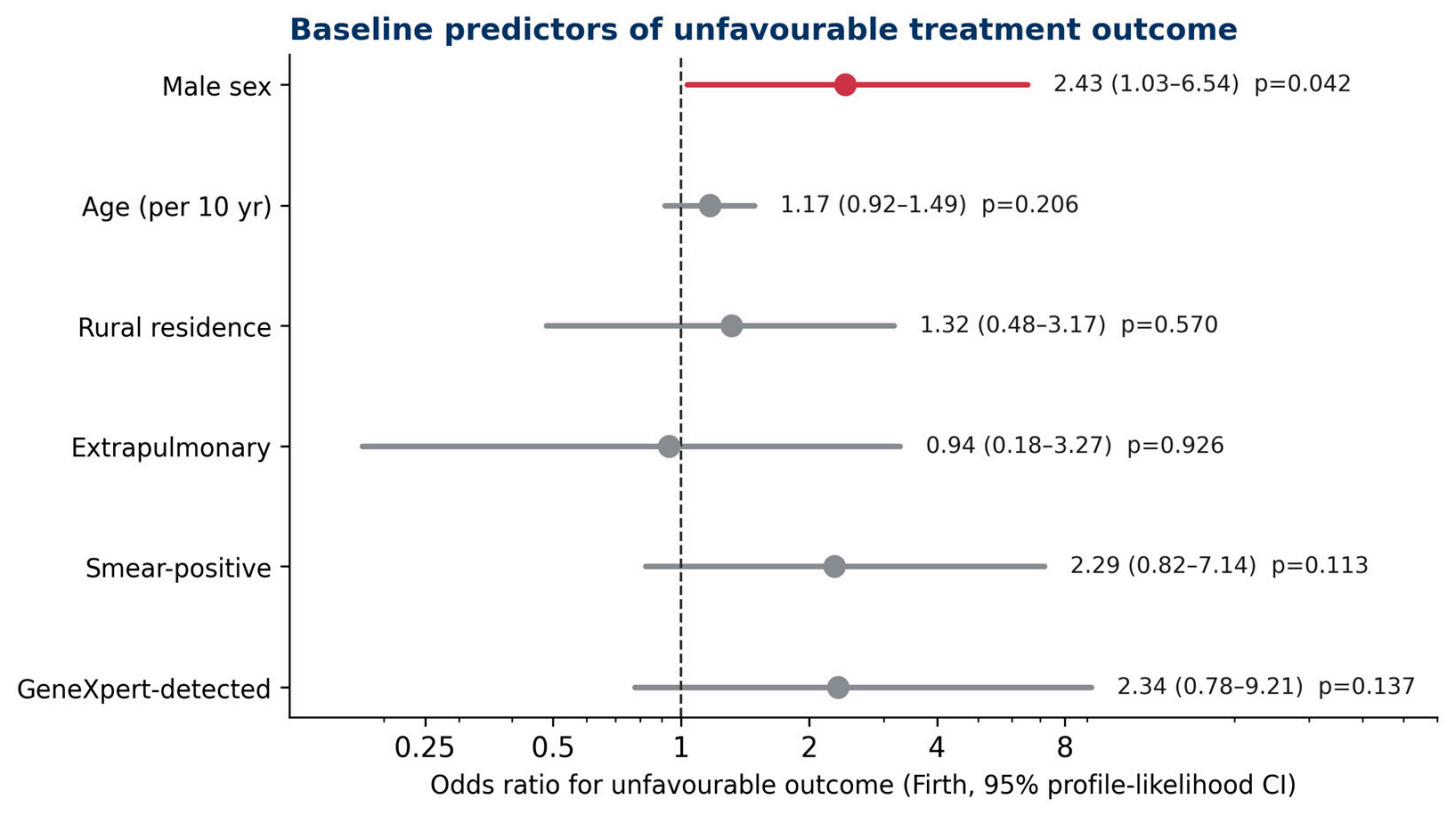
Univariate baseline predictors of unfavourable outcome (Firth OR, 95% profile-likelihood CI). Only male sex reached significance.

Among the 308 patients with a determinate outcome (27 unfavourable events), male sex was the only baseline factor associated with unfavourable outcome on univariate Firth regression (OR 2.43, 95% CI 1.03–6.54; *p* = 0.042). Age, rural residence, extrapulmonary disease, smear positivity and GeneXpert positivity were not significantly associated (Table 4, Figure 2). In a parsimonious multivariable model adjusting for GeneXpert status, male sex was attenuated and no baseline factor remained independently significant (adjusted OR 2.70, 95% CI 0.90–10.67; *p* = 0.079). The multivariable analysis included 242 patients and only 17 unfavorable events; consequently, the adjusted estimates were imprecise and should be interpreted cautiously. Excluding deaths from the unfavourable composite did not materially change these findings.

Documented non-compliance was markedly more frequent among patients with an unfavorable outcome. Among the 307 patients with a determinate outcome and available compliance information, 15 of 28 patients with documented non-compliance (53.6%) experienced an unfavorable outcome, compared with 11 of 279 compliant patients (3.9%); compliance information was unavailable for one additional patient with an unfavorable outcome. Because non-compliance was recorded during treatment and is temporally and partly definitionally related to loss to follow-up, it was analyzed as a treatment-process indicator rather than as a baseline prognostic factor. Accordingly, this association should not be interpreted as evidence of an independent causal effect. Instead, documented non-compliance should be considered an important operational marker identifying patients who may require intensified adherence support and follow-up.

### 3.5. Hospitalization During Treatment (Secondary)

Hospitalization during ambulatory treatment was required in 24 of 483 patients (5.0%). Adverse drug reactions were strongly associated with hospitalization (Firth OR 5.95, 95% CI 2.55–13.72; *p* < 0.001), indicating that treatment-related toxicity was the principal driver of inpatient referral. No baseline demographic or microbiological variable was significantly associated with hospitalization.

## 4. Discussion

This real-world retrospective cohort study evaluated the ambulatory management of drug-susceptible tuberculosis during the COVID-19 pandemic within a tertiary-care-linked dispensary network in Bucharest, Romania. Several findings are particularly relevant. First, 281 of 545 patients achieved cure or treatment completion, corresponding to a cohort-level WHO-defined treatment-success rate of 51.6%. Importantly, this relatively low programmatic proportion was largely driven by incomplete outcome ascertainment: 237 patients (43.5%) were classified as not evaluated, whereas only 27 patients (5.0%) had a documented unfavourable outcome. Among the 308 patients with a determinate end-of-treatment outcome, 281 (91.2%) achieved treatment success; however, this conditional proportion should not be interpreted as the treatment-success rate for the entire cohort. Second, no robust baseline demographic, clinical, or microbiological predictor of unfavourable outcome remained statistically significant after adjustment. Third, documented non-compliance was markedly more frequent among patients with unfavourable outcomes, although it should be interpreted as a treatment-process and operational marker rather than as an independent causal predictor. Finally, hospitalization during ambulatory treatment was uncommon among patients with available hospitalization information, while adverse drug reactions were strongly associated with inpatient referral. Because patients were selected for ambulatory management according to clinical appropriateness and no contemporaneous inpatient comparator was available, these findings primarily support the operational feasibility of this model and cannot establish comparative effectiveness or superiority over hospital-based care.

These observations should be interpreted within the longer-term evolution of tuberculosis care in Romania. Historically, TB management relied substantially on hospitalization, particularly during treatment initiation, reflecting concerns regarding transmission, clinical monitoring, treatment adherence, disease severity, and social vulnerability. Nevertheless, international tuberculosis elimination frameworks increasingly support patient-centred and decentralized models of care when clinically appropriate [11]. In addition, systematic evidence comparing hospital- and ambulatory-based management, particularly in multidrug-resistant tuberculosis settings, has demonstrated the potential role of outpatient care when adequate clinical monitoring, treatment support, and follow-up are ensured [12]. In Romania, the transition toward patient-centred ambulatory TB care had already begun before and during the early phase of the COVID-19 pandemic. A pilot project conducted within the Marius Nasta Institute dispensary network demonstrated the feasibility of outpatient diagnosis, treatment, and monitoring and formed part of a broader transition away from predominantly hospital-centred TB management [13]. Therefore, the ambulatory pathway evaluated in the present study should not be regarded solely as a temporary response to COVID-19, although pandemic-related pressure on specialized respiratory hospitals substantially accelerated its implementation.

The 2020–2022 study period should also be considered within the longer-term trajectory of Romanian TB treatment outcomes. Analysis of National Tuberculosis Programme data from 2015–2017 showed successful treatment in 84.3% of non-rifampicin-resistant TB cases among patients without the specifically analysed vulnerable-risk profiles [14]. The Romanian National Strategy for Tuberculosis Control subsequently reported a 2018 treatment-success baseline of 84.75% among new bacteriologically confirmed pulmonary drug-susceptible TB cases [15]. During the pandemic, ECDC/WHO surveillance reported treatment success of 81.6% among new and relapse TB cases notified in Romania in 2020 [16]. More recent surveillance indicates recovery toward pre-pandemic levels, while the most recent Romania country profile reports treatment success of 84.0% among new culture-positive pulmonary TB cases notified in 2023 [17,18]. Although these national surveillance cohorts are not directly comparable with the selected ambulatory population evaluated in the present study, they provide important temporal context and do not suggest a sustained national deterioration in TB treatment outcomes before, during, or after the COVID-19 pandemic.

Against this national background, the relatively low cohort-level treatment-success proportion observed in our study appears to reflect primarily incomplete local outcome ascertainment rather than a high frequency of documented treatment failure. Of the 545 patients included, 237 (43.5%) were classified as not evaluated, compared with only 17 patients lost to follow-up, five deaths, and five treatment failures. Among patients with an assigned determinate outcome, 91.2% achieved treatment success; however, this conditional proportion cannot substitute for the cohort-level programmatic indicator. Furthermore, patients with and without determinate outcomes were not entirely comparable, as extrapulmonary TB was significantly more frequent among not-evaluated patients. These findings suggest that missing outcome information was at least partly informative and identify outcome documentation, interfacility information exchange, and continuity of follow-up as important areas for improvement in ambulatory TB programs.

The analysis of baseline characteristics should similarly be interpreted with caution. Male sex was associated with unfavourable outcome in univariable Firth regression, but the association was attenuated after adjustment for GeneXpert MTB/RIF status. Age, area of residence, disease localization, smear positivity, and GeneXpert positivity were not significantly associated with unfavourable outcome. Importantly, only 27 unfavourable events occurred among patients with determinate outcomes, and the multivariable model included only 17 unfavourable events among 242 patients, resulting in limited statistical power and relatively wide confidence intervals. Consequently, the absence of statistically significant associations should not be interpreted as evidence that these variables lack prognostic relevance, but rather that their independent effects could not be reliably demonstrated in this event-limited dataset. Recent prospective evidence from a large multicentre cohort of patients with drug-susceptible pulmonary tuberculosis identified older age, male sex, lower educational attainment, undernutrition, and alcohol use as independent predictors of unfavourable treatment outcomes [19]. Complementary evidence from Romania and Ukraine has also demonstrated that unfavourable outcomes in drug-susceptible TB may be associated with factors including older age, chronic kidney disease, radiographic cavitation, underweight status, and persistent laboratory abnormalities, highlighting the importance of comprehensive clinical and nutritional risk assessment [20]. Several of these potentially relevant clinical and social determinants were unavailable in our dataset, further emphasizing the need for more comprehensive risk characterization in future prospective studies.

Treatment adherence nevertheless remains a central operational issue in decentralized TB care. Documented non-compliance was markedly more frequent among patients experiencing unfavourable outcomes. Because non-compliance was recorded during treatment and is temporally and partly definitionally related to treatment interruption and loss to follow-up, its association with unfavourable outcome should not be interpreted as demonstrating an independent causal effect. Rather, it represents an operational marker that may identify patients requiring earlier and more intensive adherence support. Recent systematic evidence evaluating digital adherence technologies suggests that approaches such as video-observed treatment, smartphone-based interventions, electronic medication monitors, and other digital tools may facilitate more patient-centred treatment supervision and improve the efficiency of adherence support, although effects on clinical outcomes remain heterogeneous and the certainty of evidence varies [21]. In parallel, a systematic review and meta-analysis of psychosocial support interventions found evidence supporting the integration of psychosocial approaches within people-centred TB care, particularly given the multidimensional barriers that patients may face during treatment [22]. These findings reinforce the importance of structured adherence assessment, patient education, early identification of treatment interruption, psychosocial evaluation, and individualized support within ambulatory TB programs.

Hospitalization during ambulatory treatment was required in only 24 of 483 patients with available information, corresponding to 5.0%. Adverse drug reactions were strongly associated with hospitalization, emphasizing that decentralization must be accompanied by effective safety mechanisms, systematic toxicity surveillance, timely clinical reassessment, and rapid access to inpatient care when clinically required. The relatively low hospitalization frequency observed in this cohort supports the operational feasibility of outpatient management among appropriately selected patients. Nevertheless, it should not be interpreted as demonstrating equivalent or superior safety compared with hospital-based care because no contemporaneous inpatient comparator group was available. This distinction is important because ambulatory care should complement, rather than replace, timely hospitalization in patients with severe disease, important complications, significant treatment toxicity, or other clinical indications for inpatient management.

The role of ambulatory TB services should also extend beyond treatment completion and microbiological response toward recognition and management of post-tuberculosis morbidity. Post-tuberculosis lung disease is increasingly recognized as an important consequence of successfully treated pulmonary TB and may include persistent structural lung damage, airway disease, pulmonary vascular abnormalities, respiratory symptoms, and impaired lung function [23]. A systematic review and meta-analysis including spirometry data from 14,621 individuals demonstrated a substantial burden of persistent lung-function impairment following pulmonary tuberculosis, further supporting the need for respiratory assessment in selected TB survivors [24]. Extrapulmonary and systemic morbidity should also be considered. A recent systematic review and meta-analysis demonstrated a relevant coexistence of cardiovascular disease among adults with tuberculosis, highlighting the broader cardiopulmonary implications of TB and the potential value of integrated long-term clinical surveillance [25]. Romanian patient-reported outcome data have additionally demonstrated that tuberculosis can affect multiple dimensions of quality of life in both drug-susceptible and drug-resistant TB populations, further supporting the incorporation of patient-centred outcomes into longitudinal follow-up [26]. Taken together, these observations expand the rationale for ambulatory follow-up beyond bacteriological cure alone and support assessment of persistent respiratory symptoms, functional impairment, obstructive or restrictive ventilatory abnormalities, cardiovascular comorbidity, and quality of life when clinically indicated.

The present findings also extend previous Romanian experience with ambulatory TB management. Earlier national evidence emphasized the importance of surveillance, diagnostic accessibility, and continuity of care, particularly among vulnerable populations [14]. National program guidance provides the organizational framework for TB diagnosis, treatment, monitoring, and follow-up [27], while previous Romanian pilot initiatives demonstrated the feasibility of transitioning selected patients from predominantly hospital-based toward outpatient management [13,28]. Evidence from Eastern Europe similarly suggests that reducing reliance on hospitalization may generate public-health and economic benefits when community services, adherence support, and appropriate referral pathways are available [29]. The present cohort is distinctive because this model was implemented during a period of exceptional health-system pressure, when specialized respiratory facilities were simultaneously required to manage large numbers of patients with SARS-CoV-2 infection.

Successful decentralization, however, requires more than simply avoiding hospitalization. Patient education, social assessment, adherence support, accessible clinical follow-up, effective communication between treatment facilities, and structured pathways for the management of acute and long-term complications are essential. Recent systematic evidence evaluating community-based TB programs supports the potential value of interventions including community health-worker treatment supervision, family-supported treatment, electronic adherence monitoring, and mobile communication strategies in improving treatment delivery and reducing barriers to care [30]. Similarly, a scoping review of patient-centred TB interventions identified education, psychosocial and socioeconomic support, targeted interventions for patients at risk of loss to follow-up, and decentralization of care as important components of a more holistic approach to TB management [31]. Patient and community acceptance must also be considered, particularly because concerns regarding transmission, household exposure, stigma, and understanding of TB may influence acceptance of outpatient management [32]. Appropriate counselling of patients and household contacts, together with clear infection-control guidance, therefore remains an integral component of safe and sustainable ambulatory treatment.

Infection prevention and control represent another essential component of this model. Previous modelling evidence has suggested that reducing unnecessary hospitalization may limit opportunities for nosocomial transmission when accompanied by appropriate administrative, environmental, and personal protective measures [33]. The present study did not evaluate household transmission, contact-investigation outcomes, or transmission within healthcare environments; therefore, no conclusions regarding the epidemiological effect of ambulatory management can be drawn. Future prospective studies should incorporate these outcomes alongside treatment success, safety, adherence, quality of life, functional recovery, patient-reported outcomes, and post-TB morbidity.

Finally, the expansion of ambulatory TB care during 2020–2022 should be interpreted within the broader reorganization of respiratory healthcare during the COVID-19 pandemic. Previous work from the same tertiary respiratory institution documented the substantial clinical demands associated with SARS-CoV-2-related inpatient care during this period [34]. International evidence subsequently demonstrated that the effect of pandemic-related disruption on TB treatment outcomes was heterogeneous across countries and regions. In an analysis of 49 high-TB-burden countries, treatment outcomes during 2020–2021 did not show uniform global deterioration; however, higher-than-expected treatment failure and mortality were observed in some analyses, while substantial regional and country-level differences remained evident [35]. These findings support cautious interpretation of pandemic-era TB outcomes and emphasize the importance of local healthcare organization and continuity of treatment services. Against this background, strengthening ambulatory pathways for clinically appropriate patients with DS-TB represented a pragmatic mechanism for maintaining essential TB services while preserving inpatient capacity.

Taken together, the present findings support the operational feasibility of structured ambulatory DS-TB management within an established tertiary-care-linked dispensary network, while also identifying important limitations in outcome ascertainment and continuity of follow-up. The findings should not be interpreted as evidence of superiority over hospital-based care. Future prospective multicentre studies should incorporate standardized eligibility criteria, comprehensive clinical, nutritional, and social risk assessment, more complete and interoperable outcome ascertainment, contemporaneous comparator groups, detailed adherence measures, transmission-related outcomes, post-TB pulmonary and cardiovascular morbidity, patient-reported outcomes, and formal cost-effectiveness analyses. Such studies would help determine which patients are most appropriately managed through ambulatory pathways and which components of decentralized TB care are most important for maintaining treatment continuity, safety, and long-term patient-centred outcomes.

## 5. Limitations

This study has several limitations that should be considered when interpreting the findings. First, its retrospective observational design limits causal inference and depends on the completeness and accuracy of routinely collected registry and medical-record data. Most importantly, end-of-treatment outcome ascertainment was incomplete: 237 of 545 patients (43.5%) were classified as not evaluated. Consequently, the cohort-level treatment-success rate was 51.6%, while 43.5% of patients were classified as not evaluated. Among patients with a determinate outcome, 91.2% achieved treatment success; however, this conditional proportion should not be interpreted as representative of the entire ambulatory cohort. Moreover, extrapulmonary TB was significantly more frequent among patients without a determinate outcome than among those with complete outcome ascertainment, suggesting that missingness was at least partly informative rather than completely random. Therefore, the complete-case treatment-success estimate should not be interpreted as directly representative of the entire ambulatory cohort.

Second, the study was conducted in TB dispensaries affiliated with a single tertiary referral center in Bucharest, which may limit the generalizability of the findings to other regions of Romania or to healthcare systems with different organizational structures, patient populations, and access to outpatient TB services. In addition, patients selected for ambulatory management may have been clinically more stable than those requiring hospitalization at diagnosis, introducing potential selection bias. The absence of a contemporaneous inpatient comparison group further prevents direct assessment of the relative effectiveness, safety, or cost-effectiveness of ambulatory versus hospital-based TB care. Accordingly, the present findings should be interpreted primarily as evidence supporting the feasibility of structured ambulatory DS-TB management in this setting rather than as evidence of its superiority over inpatient care. In addition, because the patient-level analytical cohort was intentionally restricted to 2020–2022, the present study cannot directly determine whether local treatment-outcome patterns differed before or after the COVID-19 period. National longitudinal surveillance data were therefore used to provide temporal context but should not be interpreted as a directly matched comparator population.

Third, data completeness varied across several clinical and microbiological variables, resulting in different denominators for disease localization, smear microscopy, GeneXpert MTB/RIF testing, hospitalization, and other analyses. This reflects the real-world nature of the dataset but may have introduced additional selection bias and reduced comparability between sub-analyses. The database also lacked detailed information on several potentially important clinical and social determinants of treatment outcome, including HIV status, diabetes mellitus, smoking, alcohol use, homelessness, socioeconomic vulnerability, nutritional status, body mass index, radiological disease severity, cavitary or bilateral pulmonary involvement, and baseline symptom burden. Residual confounding from these unmeasured factors therefore cannot be excluded.

Fourth, the number of unfavorable treatment outcomes was small, with only 27 events among 308 patients with a determinate outcome. Although Firth penalized-likelihood regression was used to reduce small-sample bias and address potential separation, the limited number of events constrained the complexity of the multivariable analysis and resulted in relatively wide confidence intervals. The finding that male sex was associated with unfavorable outcome in univariable analysis but not after adjustment should therefore be interpreted cautiously, and the absence of statistically significant associations for other baseline characteristics should not be regarded as evidence of absence of clinically meaningful effects. Similarly, documented non-compliance was analyzed as a treatment-process indicator rather than as a baseline prognostic factor because it occurs during treatment and is temporally related to outcomes such as treatment interruption and loss to follow-up.

Finally, the study did not evaluate household or nosocomial TB transmission, contact-investigation outcomes, infection-control performance, patient satisfaction, health-related quality of life, stigma, or direct and indirect costs. These outcomes are important when evaluating the broader public-health impact, acceptability, and sustainability of ambulatory TB care. Future prospective multicenter studies should therefore aim for more complete outcome ascertainment, include contemporaneous comparator groups, collect standardized clinical and social risk factors, and incorporate transmission-related outcomes, patient-reported measures, and formal cost-effectiveness analyses.

## 6. Conclusions

In this real-world retrospective cohort, structured ambulatory care for drug-susceptible tuberculosis during the COVID-19 pandemic was feasible within a tertiary-care-linked dispensary network in Bucharest. Among patients with a determinate end-of-treatment outcome, treatment success was high, while hospitalization during treatment was uncommon. However, the substantial proportion of patients classified as not evaluated limits the precision and generalizability of the overall treatment-success estimate and requires cautious interpretation of the complete-case results.

No robust baseline demographic, clinical, or microbiological predictor of unfavorable outcome was identified after adjustment, although male sex was associated with unfavorable outcome in univariable analysis. By contrast, documented non-compliance was strongly associated with unfavorable outcome as a treatment-process indicator, emphasizing the importance of continuous adherence monitoring and patient support throughout ambulatory treatment. Adverse drug reactions were strongly associated with hospitalization, highlighting the need for systematic toxicity surveillance, rapid clinical reassessment, and clear referral pathways within outpatient TB programs.

Overall, these findings provide evidence that structured, patient-centered ambulatory DS-TB care was feasible to implement in this Romanian tertiary-care-linked setting when outpatient monitoring, adherence support, adverse-event management, and access to hospitalization were available. Nevertheless, because of the retrospective design, incomplete outcome ascertainment, potential selection bias, and absence of a contemporaneous inpatient comparator group, the present study should be interpreted as evidence of feasibility rather than superiority of ambulatory over hospital-based care. Future prospective multicenter studies with more complete outcome ascertainment, standardized clinical and social risk assessment, comparator groups, transmission-related outcomes, patient-reported measures, and formal cost-effectiveness analyses are needed to determine the long-term effectiveness, safety, and scalability of this model beyond the pandemic context.

## Figures and Tables

**Table 1:** Baseline characteristics and treatment-process variables of the drug-susceptible TB cohort (n = 545).

Characteristic	Category	Value
Sex	Male	306 (56.1%)
	Female	239 (43.9%)
Age, years	Mean ± SD	44.8 ± 16.0
Residence	Urban	443 (81.6%)
	Rural	100 (18.4%)
Localization *	Pulmonary	278 (80.6%)
	Extrapulmonary	61 (17.7%)
	Pulmonary + extrapulmonary	6 (1.7%)
Smear microscopy *	Positive	183 (52.1%)
	Negative	168 (47.9%)
GeneXpert MTB/RIF *	Detected	278 (68.6%)
	Not detected	127 (31.4%)
Non-compliance (process)	Yes	67 (12.3%)
Adverse drug reactions (process)	Yes	71 (13.0%)
Hospitalization (process)	Yes	24 (5%)

* Percentages for localization, smear microscopy, GeneXpert MTB/RIF, and hospitalization were calculated using patients with the corresponding variable recorded (localization n = 345, smear microscopy n = 351, GeneXpert MTB/RIF n = 405, hospitalization n = 483). Missing observations were excluded from variable-specific percentage calculations.

**Table 2:** End-of-treatment outcomes (WHO 2021 definitions), n = 545.

Outcome	n	% of Cohort
Cured	164	30.1
Treatment completed	117	21.5
Treatment success (total)	281	51.6
Loss to follow-up	17	3.1
Died	5	0.9
Treatment failure	5	0.9
Unfavourable outcome (total)	27	5.0
Not evaluated	237	43.5

Treatment success was calculated using the entire DS-TB cohort as the denominator according to the WHO programmatic treatment-outcome framework: 281/545 = 51.6%. Among the 308 patients with a determinate end-of-treatment outcome, 281 (91.2%) were successfully treated; this conditional proportion is reported descriptively and does not represent the overall cohort treatment-success rate.

**Table 3:** Determinate-outcome versus not-evaluated patients.

Variable	Determinate (n = 308)	Not Evaluated (n = 237)	*p*
Age, median (years)	41	44	0.056
Male sex	183/308 (59%)	123/237 (52%)	0.082
Rural residence	58/307 (19%)	42/236 (18%)	0.823
Extrapulmonary	24/191 (13%)	37/154 (24%)	0.007
Smear-positive	94/190 (49%)	89/161 (55%)	0.286
GeneXpert-detected	158/242 (65%)	120/163 (74%)	0.081

Mann–Whitney U test (age); Fisher’s exact test (categorical). Denominators vary with data completeness.

**Table 4:** Predictors of unfavourable treatment outcome (Firth penalized logistic regression, 95% profile-likelihood CI).

Predictor	OR/aOR (95% CI)	*p*
Male sex	2.43 (1.03–6.54)	0.042
Age (per 10 yr)	1.17 (0.92–1.49)	0.206
Rural residence	1.32 (0.48–3.17)	0.570
Extrapulmonary	0.94 (0.18–3.27)	0.926
Smear-positive	2.29 (0.82–7.14)	0.113
GeneXpert-detected	2.34 (0.78–9.21)	0.137
Multivariable (male sex + GeneXpert)		
Male sex	2.70 (0.90–10.67)	0.079
GeneXpert-detected	2.16 (0.72–8.57)	0.181

Reference categories: female sex, urban residence, pulmonary disease, smear-negative, GeneXpert not detected. Univariate n and events vary with completeness; multivariable n = 242, events = 17.

## Data Availability

The data supporting the findings of this study are available from the corresponding author upon reasonable request, subject to institutional approval and applicable data-protection regulations.
